# Association of Chest Pain and Risk of Cardiovascular Disease with Coronary Atherosclerosis in Patients with Inflammatory Joint Diseases

**DOI:** 10.3389/fmed.2015.00080

**Published:** 2015-11-10

**Authors:** Silvia Rollefstad, Eirik Ikdahl, Jonny Hisdal, Tore Kristian Kvien, Terje Rolf Pedersen, Anne Grete Semb

**Affiliations:** ^1^Preventive Cardio-Rheuma Clinic, Department of Rheumatology, Diakonhjemmet Hospital, Oslo, Norway; ^2^Section of Vascular Investigations, Oslo University Hospital Aker, Oslo, Norway; ^3^Department of Rheumatology, Diakonhjemmet Hospital, Oslo, Norway; ^4^Centre of Preventive Medicine, Oslo University Hospital, Oslo, Norway; ^5^Faculty of Medicine, University of Oslo, Oslo, Norway

**Keywords:** atherosclerosis, chest pain, cardiovascular diseases, inflammatory joint diseases, risk factors

## Abstract

**Objectives:**

The relation between chest pain and coronary atherosclerosis (CA) in patients with inflammatory joint diseases (IJD) has not been explored previously. Our aim was to evaluate the associations of the presence of chest pain and the predicted 10-year risk of cardiovascular disease (CVD) by use of several CVD risk algorithms, with CA verified by multidetector computed tomography (MDCT) coronary angiography.

**Methods:**

Detailed information concerning chest pain and CVD risk factors was obtained in 335 patients with rheumatoid arthritis and ankylosing spondylitis. In addition, 119 of these patients underwent MDCT coronary angiography.

**Results:**

Thirty-one percent of the patients (104/335) reported chest pain. Only six patients (1.8%) had atypical angina pectoris (pricking pain at rest). In 69 patients without chest pain, two thirds had CA, while in those who reported chest pain (*n* = 50), CA was present in 48.0%. In a logistic regression analysis, chest pain was not associated with CA (dependent variable) (*p* = 0.43). About 30% (Nagelkerke *R*^2^) of CA was explained by any of the CVD risk calculators: Systematic Coronary Risk Evaluation, Framingham Risk Score, or Reynolds Risk Score.

**Conclusion:**

The presence of chest pain was surprisingly infrequently reported in patients with IJD who were referred for a CVD risk evaluation. However, when present, chest pain was weakly associated with CA, in contrast to the predicted CVD risk by several risk calculators which was highly associated with the presence of CA. These findings suggest that clinicians treating patients with IJD should be alert of coronary atherosclerotic disease also in the absence of chest pain symptoms.

## Introduction

Patients with inflammatory joint diseases (IJD) have an increased risk of cardiovascular disease (CVD) (1–3). Despite this well-established knowledge, implementation of CVD risk evaluation as a part of standard care in patients with IJD is deficient (4, 5). Chest pain is a vital symptom of coronary atherosclerotic disease, and its presence is addressed as a natural part of a CVD risk assessment. Chest pain in patients with IJD may be related to angina pectoris or to the rheumatic disease itself, and it can be clinically difficult to distinguish between the two for both the patient and the physician. The relation between chest pain and coronary disease in patients with rheumatoid arthritis (RA) and ankylosing spondylitis (AS) has not been explored previously.

Angina pectoris is a symptom of myocardial ischemia caused by coronary atherosclerosis (CA) and may be diagnosed by several techniques. The selective coronary angiography procedure has become the gold standard for diagnosis of CA. This is an invasive, expensive, and resource-demanding procedure. Multidetector computed tomography (MDCT) coronary angiography is another imaging technique for identifying CA (6). Although both methods share the risks of radiation exposure and adverse reactions to contrast medium, MDCT coronary angiography has certain advantages over selective coronary angiography in that it is less costly, more time efficient, and entails a lower risk of CVD complications due to its non-invasiveness. The clinical utility of MDCT coronary angiography in predicting important CVD outcomes has been evaluated in several studies (7–9). The method is suitable for detection of CA in patients with moderate risk of CVD (10), and due to the high negative predictive value, it is particularly useful for excluding coronary stenosis (11).

The aim of the present report was to characterize and compare the various types of chest pain in patients with RA and AS who were referred for a CVD risk evaluation. Furthermore, we sought to evaluate the associations of the presence of chest pain and the predicted 10-year risk of CVD by use of several CVD risk algorithms, with CA (verified by MDCT coronary angiography).

## Materials and Methods

Patients with RA and AS were referred from a rheumatology outpatient clinic or from general practitioners, for CVD risk evaluation at the Preventive Cardio-Rheuma Clinic at Diakonhjemmet Hospital, Oslo, during the period March 2009–July 2012. Details concerning this preventive cardio-rheuma clinic and the approach to identify CVD risk factors, comorbidities, and biomarkers have been described previously (12, 13). Referred patients received a CVD questionnaire by mail, including detailed questions regarding chest pain. At the clinical consultation, the answers in the patient self-reported questionnaire were verified by a cardiologist (AGS). The various types of chest pain were grouped into classical and non-classical angina pectoris. Non-classical chest pain was defined as pricking and cutting pain during rest and only lasting for seconds. Classical coronary chest pain was defined as any of the following: tightness, squeezing, pressure, compression, heaviness, band around the chest, and nausea lasting for 1 min or more. The pain should be localized centrally and/or on the side(s) of thorax, radiating to throat/jaw or out in the arms/back and related to physical activity, as walking up hill/steps, walking indoors/flat, or while performing housework. In order to distinguish possible CA that would demand urgent invasive investigations, from stable coronary artery disease, patients were also asked questions to clarify the duration, frequency, persistence, and activity level needed to trigger the pain. Our cohort consisted of patients with low-intensity chest pain who were not in need of immediate assessment with selective coronary angiography.

Patients with RA and AS at moderate to high risk of CVD and/or presence of chest pain were referred to MDCT coronary angiography.

This is an observational report from a quality assurance register. Ethical approval and informed patient consent was therefore not required. The data collection/publication has been recommended and approved by the Oslo University Hospitals’ Office of Privacy and Data Protection (2011/7318).

### MDCT Coronary Angiography

The MDCT coronary angiography examination was performed at the Oslo University Hospital, Ullevaal. Two methods were used: first, spiral coronary artery MDCT, where 64 detectors were employed. This method is advantageous when performing reconstruction of the coronary arteries in several planes, including a three-dimensional plane. Furthermore, images of the coronary arteries may be reconstructed during all stages of the electrocardiogram (ECG) cycle. Second, the step and shoot technique was used. This is an ECG-gated MDCT coronary angiography method to reduce movement artifacts during the cardiac contraction cycle. To obtain optimal image quality with minimal movement artifacts, the heart rate should be <60 beats/min. Therefore, the patients were given a beta blocker 2 h before the procedure. The step and shoot MDCT coronary angiography is only performed during certain phases of the heart cycle, which means that the radiation exposure can be reduced by as much as 80% compared to spiral CT. In our center, the step and shoot procedure reduced radiation exposure from 9–14 to 3 mSv. Therefore, the step and shoot MDCT coronary angiography was the preferred method, but when heart rate <60 beats/min was not obtained, spiral MDCT coronary angiography was performed.

Iodine-based intravenous contrast medium was used in both MDCT methods, and all patients were asked if they had allergies and, specifically, if they had experienced an allergic reaction against contrast medium previously. Patients with previous allergic reactions to contrast medium were given anti-histamines and/or corticosteroids before the MDCT coronary angiography examination. Contrast medium may affect kidney function. All patients with indication for MDCT coronary angiography in this report had normal kidney function (estimated glomerular filtration rate ≥60 ml/min). Diabetic patients using metformin were asked to abstain from this medication for 48 h after the contrast administration. The kidney function was evaluated after the procedure, and in specific cases, metformin medication was not restarted until the kidney function was normalized.

Coronary atherosclerosis was diagnosed by the interventional cardiologist/radiologist. When the quality of the MDCT coronary angiography images was inadequate, or if the examination revealed suspicion of a significant coronary stenosis, selective coronary angiography was performed. In cases of coronary stenosis, individual evaluation was conducted regarding interventions such as percutaneous coronary intervention (PCI), open-heart surgery with coronary artery bypass grafting (CABG), or for optimization of cardioprotective medical treatment.

### CVD Risk Algorithms

The risk of future CVD was calculated by applying the following CVD risk calculators: (1) the Systematic COronary Risk Evaluation (SCORE) (14), which predicts the 10-year risk of a fatal atherosclerotic event and includes the following risk factors: age, sex, smoking status, systolic blood pressure (BP), and total cholesterol (TC); (2) the Framingham Risk Score (FRS) (15) incorporating age, sex, smoking status, BP, antihypertensive treatment, TC, high-density lipoprotein cholesterol (HDL-c), and diabetes; and (3) the Reynolds Risk Score (RRS) (16, 17), which contains age, sex, smoking status, BP, TC, HDL-c, C-reactive protein (CRP), and family history of premature myocardial infarction. Both the FRS and the RRS calculate the risk of a fatal or non-fatal CVD event coming 10 years.

### Statistics

The data are presented as crude data, and the results are expressed as mean ± SD and median and interquartile range (IQR) for normally and non-normally distributed characteristics, respectively. The data were compared using independent samples *t*-test, non-parametric tests, and χ^2^-tests as appropriate. A logistic regression model was constructed comparing CA versus no CA as the dependent variable in order to assess the associations with both chest pain and CVD risk factors. Instead of investigating the correlation to the CVD risk factors separately, they were examined collectively by applying the aforementioned CVD risk calculators’ estimate of future risk of a CVD event as independent variables in the logistic regression models. Due to the multicollinearity between the CVD risk calculators, separate models were constructed for each of the three CVD risk algorithms. Data analyses were performed using IBM SPSS version 20.

## Results

### Chest Pain Characteristics

**Figure 1:**
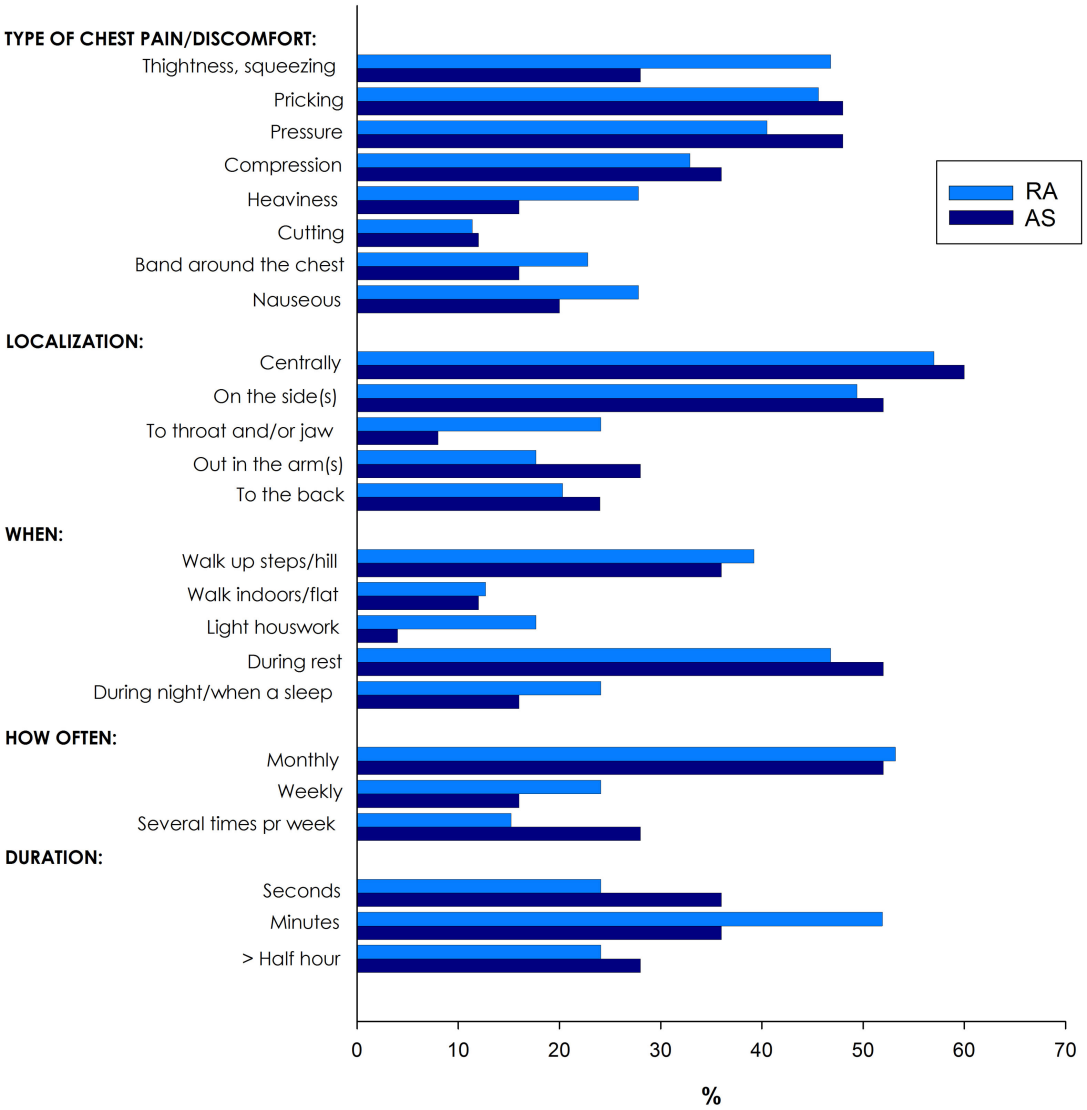
**Various types of chest pain in patients with rheumatoid arthritis (RA) and ankylosing spondylitis (AS)**.

Of the 335 patients with RA and AS referred to the Preventive Cardio-Rheuma Clinic, 104 (31.0%) patients reported chest pain (Figure 1), of which six patients experienced non-classical angina pectoris (pricking pain, occurring at rest and lasting for seconds). In general, there were no significant differences between RA and AS concerning the various types (i.e., localization and relation to physical activity), frequency, or duration of the chest pain episodes. The types of chest pain which varied the most between RA and AS patients were tightness/squeezing and heaviness, radiating to throat/jaw or related to light housework, but these differences did not reach a level of statistical significance (Figure 1).

### Chest Pain and Coronary Atherosclerosis

**Figure 2:**
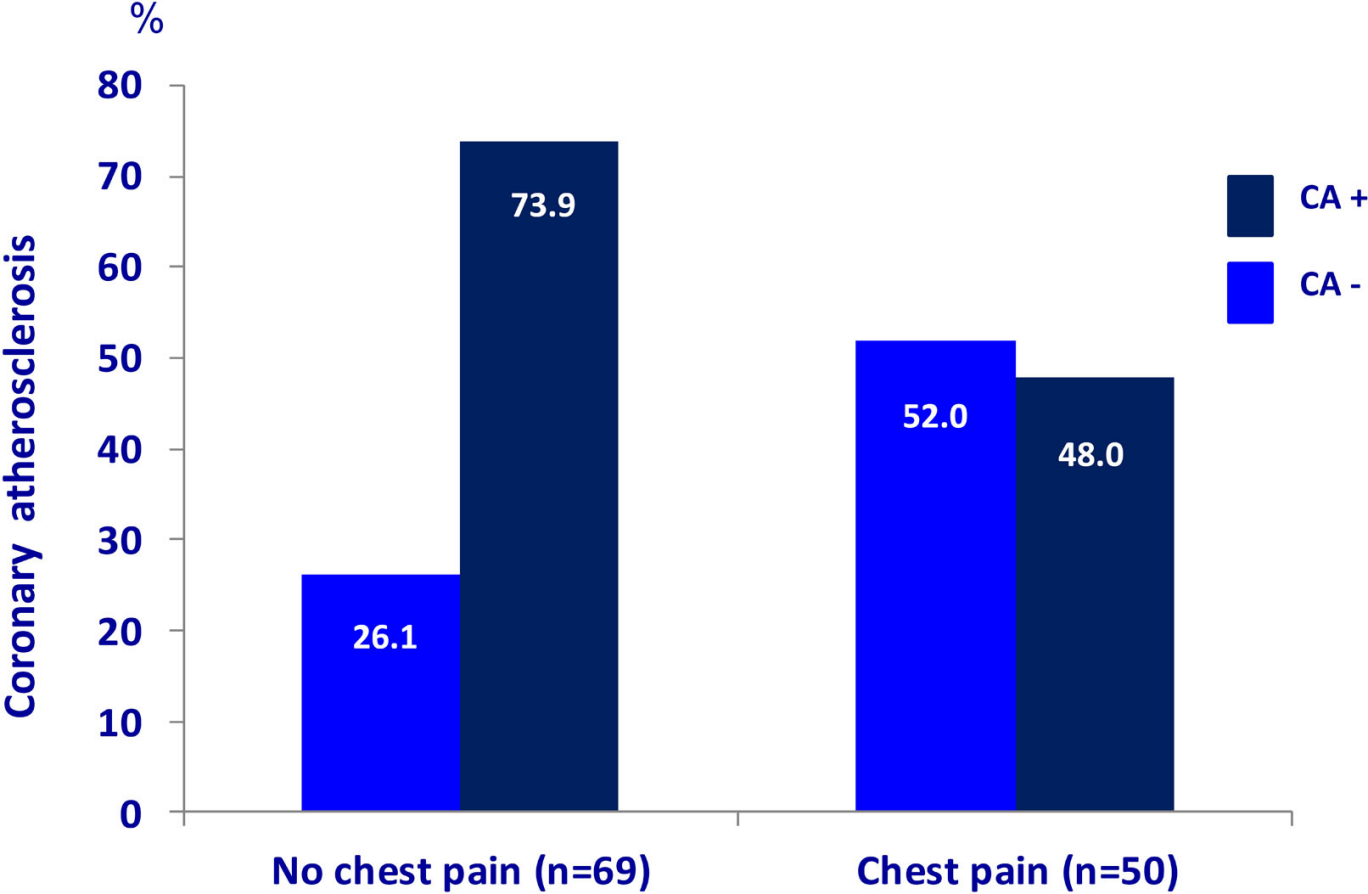
**Chest pain and coronary atherosclerosis in RA and AS**. Presence of coronary atherosclerosis (examined with multidetector computed tomography) in rheumatoid arthritis and ankylosing spondylitis patients (*n* = 119) with and without chest pain. RA, rheumatoid arthritis; AS, ankylosing spondylitis.

**Figure 3:**
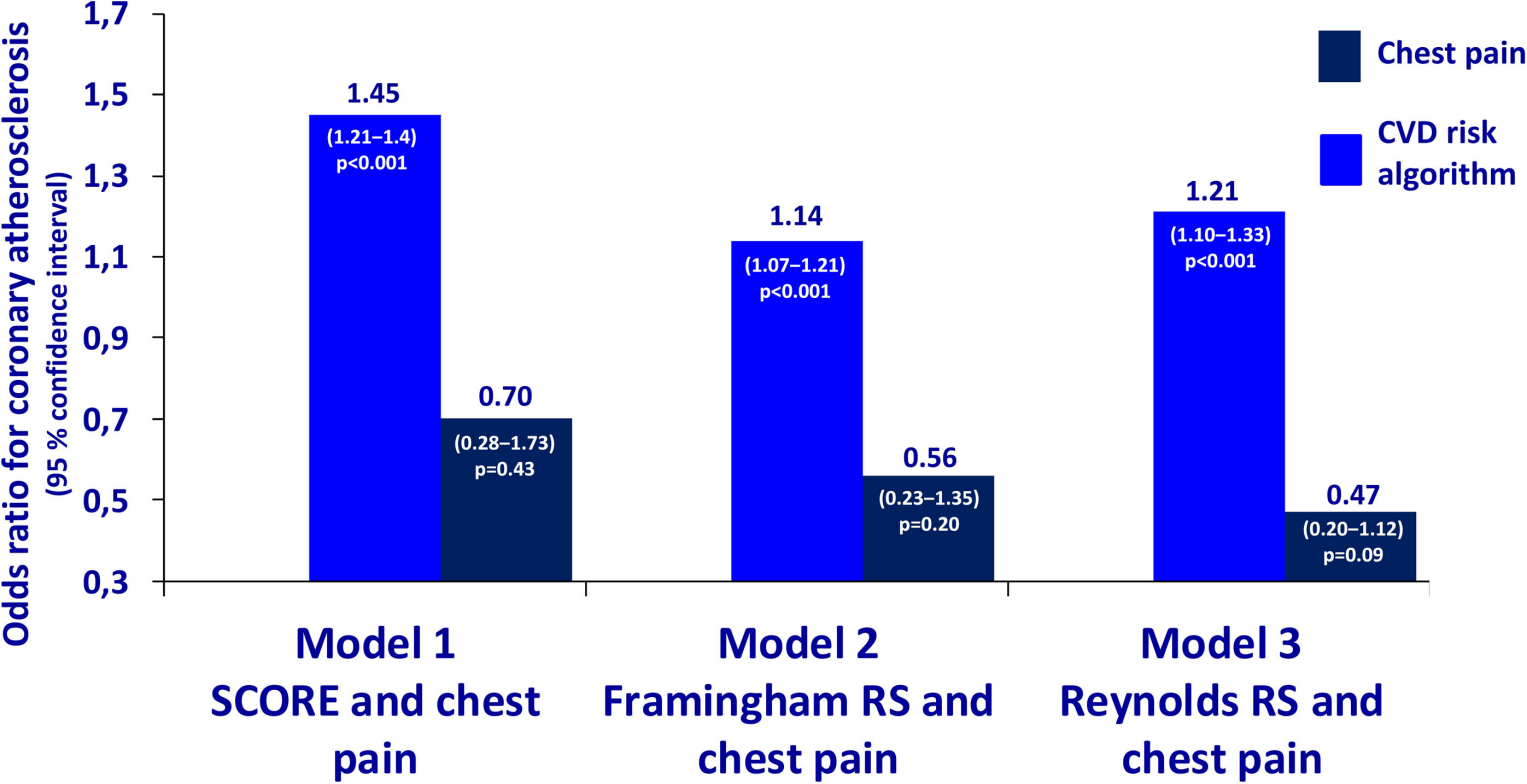
**Odds ratios for coronary atherosclerosis in RA and AS**. Association of coronary atherosclerosis with chest pain and the calculated CVD risk by several risk calculators in patients with rheumatoid arthritis and ankylosing spondylitis. Statistics: the estimates are presented as odds ratios for the presence of CA. The odds ratios are analyzed per unit increase of CVD risk calculated by SCORE, Framingham Risk Score, and Reynolds Risk Score. RA, rheumatoid arthritis; AS, ankylosing spondylitis; SCORE, systematic coronary risk evaluation; CVD, cardiovascular disease.

The characteristics of the 119 patients with RA and AS referred to MDCT coronary angiography is described in Table 1. As expected, the sex distribution was inverse in RA and AS (*p* < 0.001). Disease duration was longer in patients with AS compared to RA patients (*p* = 0.01). Furthermore, AS patients had more hypertension (*p* = 0.04) and a higher calculated risk of CVD by use of the SCORE algorithm (*p* = 0.05) compared to RA patients. On the other hand, RA patients experienced more chest pain than patients with AS (*p* = 0.04), whereas the proportion of patients with CA was comparable between the two diagnoses (*p* = 0.48). Associations between CVD risk factors and CA are presented in Table 2. Patients with CA were older (<0.001), more often had diabetes (*p* = 0.001), and had a higher CVD risk evaluated by SCORE, FRS, and RRS (*p* < 0.001 for all) compared to those without CA. In patients without chest pain, two thirds had CA, while in those reporting chest pain, approximately half of the patients had CA (Figure 2). In a logistic regression analysis with CA as the dependant variable, including chest pain and SCORE, chest pain was not associated with CA (dependent variable) (*p* = 0.43), while the calculated CVD risk by SCORE was highly associated with CA (*p* < 0.001) (Figure 3). Comparable results were revealed in logistic regression models incorporating FRS or RRS instead of the SCORE algorithm, where the calculated CVD risk was highly associated with CA (*p* = 0.20 and *p* = 0.09, respectively), whereas chest pain was not (*p* = 0.20 and *p* = 0.09, respectively). Approximately 30% (Nagelkerke *R*^2^) of CA was explained by the calculated CVD risk using all the three CVD risk calculators (data not shown).

**Table 1:** **Baseline characteristics of patients examined by MDCT coronary angiography**.

	RA	AS	*p*-value
**Number**	86 (72.3)	33 (27.7)	
Age, median (IQR)	60.0 (54.8–68.0)	59.0 (54.5–65.5)	0.56
Sex, male/female, *n* (%)	21/65 (21.4)/(75.6)	22/11 (66.7)/(33.3)	<0.001
Disease duration, years, median (IQR)	16.0 (7.0–24.8)	25.0 (15.5–31.0)	0.01
**CVD risk factors, mean ± SD**		
Smoke, *n* (%)	16 (18.6)	5 (15.2)	0.66
BMI	24.9 ± 3.6	25.0 ± 2.3	0.25
TC, mmol/L	6.13 ± 1.27	5.88 ± 0.99	0.46
HDL-c, mmol/L	1.79 ± 0.51	1.58 ± 0.47	0.06
TG, mmol/L, median (IQR)	1.13 (0.83–1.54)	1.14 (0.87–1.66)	0.66
LDL-c, mmol/L	3.71 ± 1.02	3.65 ± 0.99	0.37
Systolic BP, mmHg	139.4 ± 20.7	145.3 ± 14.7	0.17
Diastolic BP, mmHg	81.5 ± 8.7	84.6 ± 8.9	0.09
**Comorbidities, *n* (%)**			
HT	45 (52.3)	24 (72.7)	0.04
Diabetes	7 (8.1)	4 (12.1)	0.50
Chest pain	41 (47.7)	9 (27.3)	0.04
Coronary atherosclerosis	54 (62.8)	23 (69.7)	0.48
**CVD risk calculators, mean ± SD**			
SCORE	4.21 ± 3.88	5.86 ± 3.95	0.05
Framingham	15.97 ± 13.01	20.28 ± 11.85	0.09
Reynolds	7.88 ± 8.20	10.98 ± 8.06	0.08
**Biomarkers**			
ESR, mean ± SD	18.5 ± 14.5	16.0 ± 11.0	0.42
CRP, median (IQR)	3.0 (1.0–6.0)	2.5 (1.0–7.6)	0.80
**Medication, *n* (%)**			
Prednisolone	38 (44.2)	3 (9.1)	<0.001
NSAIDs	34 (39.5)	17 (51.5)	0.22
sDMARDs	48 (55.8)	7 (21.2)	0.001
bDMARDs	29 (33.7)	13 (39.4)	0.76
Statins	6 (7.0)	1 (3.0)	0.41
HT medication	21 (24.4)	8 (24.2)	0.98

*RA, rheumatoid arthritis; AS, ankylosing spondylitis; CVD, cardiovascular; BMI, body mass index; TC, total cholesterol; HDL, high-density lipoprotein cholesterol; TG, triglycerides; LDL-c, low-density lipoprotein cholesterol; BP, blood pressure; HT, hypertension; SCORE, systematic coronary risk evaluation; ESR, erythrocyte sedimentation rate; CRP, C-reactive protein; NSAIDs, non-steroid anti-inflammatory drugs; sDMARDs, synthetic disease modifying antirheumatic drugs; bDMARDs, biologic disease modifying antirheumatic drugs; MDCT, multidetector computed tomography*.

**Table 2:** **Coronary atherosclerosis in relation to cardiovascular risk factors and chest pain in patients with RA and AS**.

	Coronary atherosclerosis		*p*-value
	No	Yes	
Total patient number, *n* (%)	42 (35.3)	77 (64.7)	–
RA, *n* (%)	32 (76.2)	54 (70.1)	0.48
AS, *n* (%)	10 (23.8)	23 (29.9)	0.48
Age, mean ± SD	53.8 ± 7.1	64.1 ± 8.0	<0.001
Sex (male/female), *n* (%)	11/31 (26.2/73.8)	32/45 (41.6/58.4)	0.10
Familial CVD disease, *n* (%)	7 (16.7)	12 (15.6)	0.88
CRP, mean ± SD	1.07 ± 1.20	1.18 ± 1.17	0.63
Diabetes, *n* (%)	0 (0.0)	11 (14.3)	0.01
**CVD risk calculators, mean ± SD**			
Score	2.38 ± 2.58	5.91 ± 4.02	<0.001
Framingham Risk Score	9.92 ± 8.56	21.12 ± 13.03	<0.001
Reynolds Risk Score	4.29 ± 5.44	11.04 ± 8.55	<0.001
**CHEST PAIN**			
**Types of chest pain, *n* (%)**			
Tightness, squeezing	9 (21.4)	17 (22.1)	0.94
Pricking	13 (31.0)	11 (14.3)	0.03
Pressure	6 (14.3)	13 (16.9)	0.71
Compression	7 (16.7)	11 (14.3)	0.73
Heaviness	4 (9.5)	7 (9.1)	0.94
Cutting	1 (2.4)	1 (1.3)	0.66
Band around the chest	6 (14.3)	5 (6.5)	0.16
Nauseous	7 (16.7)	9 (11.7)	0.45
**Location, *n* (%)**			
Centrally	13 (31.0)	19 (24.7)	0.46
On the side(s)	14 (33.3)	15 (19.5)	0.09
To throat and/or jaw	2 (4.8)	8 (10.4)	0.29
Out in the arm(s)	2 (4.8)	8 (10.4)	0.29
To the back	7 (16.7)	7 (9.1)	0.22
**When, *n* (%)**			
Walk up steps/hill	9 (21.4)	13 (16.9)	0.54
Walk indoors/flat	2 (4.8)	2 (2.6)	0.53
Light housework	2 (4.8)	4 (5.2)	0.92
During rest	11 (26.2)	17 (22.1)	0.61
During night/when a sleep	6 (14.3)	5 (6.5)	0.16
**How often, *n* (%)**			
Monthly	15 (35.7)	17 (22.1)	0.11
Weekly	6 (14.3)	6 (7.8)	0.26
Several times per week	6 (14.3)	5 (6.5)	0.16
**Duration, *n* (%)**			
Seconds	7 (16.7)	7 (9.1)	0.22
Minutes	15 (35.7)	17 (22.1)	0.11
≥Half hour	7 (16.7)	4 (5.2)	0.04
**Chest pain total, *n* (%)**	24 (57.1)	26 (33.8)	0.01

*RA, rheumatoid arthritis; AS, ankylosing spondylitis; CVD, cardiovascular; CRP, C-reactive protein; SCORE, systematic coronary risk evaluation*.

### Selective Coronary Angiography

**Figure 4:**
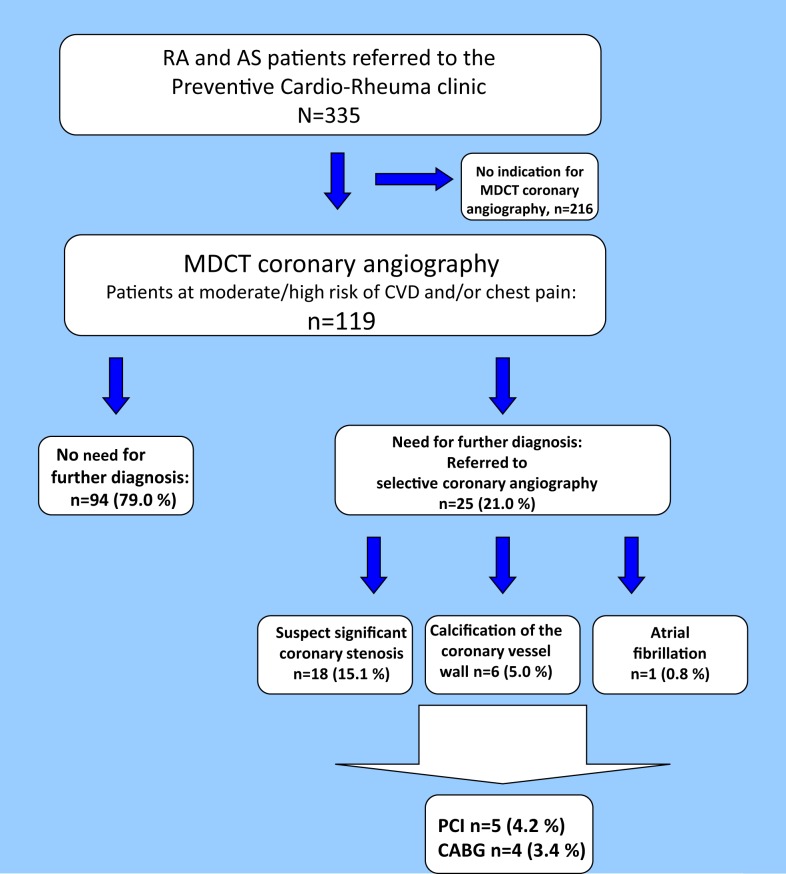
**Results of the MDCT coronary angiography**. Flowchart of patients undergoing MDCT coronary angiography. MDCT, multidetector computed tomography; CA, coronary atherosclerosis; PCI, percutaneous coronary intervention; CABG, coronary artery bypass grafting.

Twenty-five patients (21.0%) were referred to selective coronary angiography based on the results of the MDCT coronary angiography (Figure 4). The majority of the patients examined with selective coronary angiography were in need of this invasive method to confirm or disprove a significant coronary stenosis (*n* = 18; 72.0%). Furthermore, indication for selective coronary angiography was found in six patients (24.0%) due to distinct calcification that disrupted the MDCT coronary angiography images and in one patient (4.0%) who had atrial fibrillation. After the selective coronary angiography, approximately two thirds of the patients were in need of optimization of the cardioprotective medical treatment (*n* = 16; 64.0%). Indication for further invasive procedures was found in nine patients [PCI *n* = 5 (20.0%) and CABG *n* = 4 (16.0%)].

## Discussion

The presence of chest pain is routinely addressed during CVD risk evaluation. The main finding in the present report was that two thirds of the IJD patients without chest pain had CA verified by MDCT coronary angiography, which may be explained by a high prevalence of silent angina pectoris. Chest pain in patients with RA and AS was not associated with CA, but clinicians are advised to ask the patients about the presence of chest pain to reveal those with classical angina pectoris and/or at immediate need of intervention. To our knowledge, the lack of relationship between presence of chest pain and CA in patients with IJD has not previously been reported.

In the general population, silent angina pectoris has been reported to occur in 10–15% of patients with coronary heart disease (18). In the study by Erikssen et al., the rate of cardiac events was four to five times higher in persons without chest pain, but with documented myocardial ischemia by exercise ECG, than in persons with no manifest ischemic disease. The study concluded that documentation of myocardial ischemia in asymptomatic patients (no chest pain) was indicative of the presence of coronary artery disease and was associated with progression of CA in spite of the absence of angina pectoris (18). Comparable findings were reported by Evans and colleagues, showing that in RA patients with asymptomatic atherosclerosis in the carotid arteries, there was a 2.5-fold to fourfold increased risk of acute coronary syndrome compared to those without carotid artery plaques (19). Furthermore, Maradit-Kremers et al. reported that patients with RA had more silent angina pectoris and myocardial infarction compared to patients without RA (20).

In addition to visualization of CA, coronary artery calcium (CAC) score may be analyzed during the MDCT coronary angiography procedure. The value of CAC score in prediction of CVD events has been thoroughly investigated (21, 22). Nevertheless, neither the European Society of Cardiology/European Atherosclerotic Society nor the American College of Cardiology/American Heart Association CVD prevention guidelines recommend including the CAC score in the evaluation of CVD risk (23, 24). Interestingly, recently published evidence concludes that statin therapy increases the CAC score (25), which is counterintuitive considering the well-established protective effect of statin treatment on future CVD events (26). Therefore, the focus in our study was to evaluate the association of chest pain with the presence of visualized atherosclerosis and not the CAC score. Furthermore, we did not perform functional testing for myocardial ischemia in our patients, due to that patients with IJD often are not able to perform a test including cycling or a treadmill. A further limitation to our report is that a negative MDCT coronary angiography would not preclude myocardial ischemia due to small-vessel disease or coronary vasospasms, both of which could cause chest pain. However, the treatment consequences are greater for the presence of CA in the vessels evaluated by MDCT coronary angiography than for small-vessel pathology and vasospasms (23).

There is a preponderance of females with RA, and it is well documented that CVD is diagnosed at later stages in females compared to males (27). Furthermore, females in the general population have been reported to have different symptoms from CA than males (28, 29), and this is probably also the case in RA patients. Unfortunately, we were not able to analyze the sex difference aspect in relation to chest pain and CA due to low numbers and corresponding lack of power.

The spondyloarthropathies, and in particular the AS disease, comprise inflammation of joints in thorax more commonly than in RA (30). We therefore assumed that AS patients would report more chest pain than patients with RA. Surprisingly, the results from this analysis showed that the opposite was true, despite the occurrence of CA being equally frequent in the two diseases. Part of the explanation for this difference may lie in the female preponderance in RA. It has been demonstrated that female RA patients report pain to a greater extent than male RA patients (31).

When analyzing the various types of chest pain, the number of patients in each symptom group was low, and we were therefore not able to conclude on the importance of the various types of chest pain and their association with CA in patients with IJD.

We cannot exclude a selection bias in our cohort in that the referral criteria to the Preventive Cardio-Rheuma Clinic were diagnosed IJD, age between 25 and 85 years, and fulfillment of at least one of the following criteria: known CVD risk factor(s), symptoms/signs of a risk factor, familial premature CVD disease, or simply that a patient wished to undergo CVD risk evaluation. Interestingly, a recent study reported that 30% of RA patients with CVD, diabetes, or hyperlipidemia consulted primary care physicians less than annually, despite frequent visits with other health providers, as rheumatologists (32). Thus, there is a possibility that a proportion of the IJD patients at high risk of CVD were not referred to the Preventive Cardio-Rheuma Clinic. The European League Against Rheumatism recommend that CVD risk assessment should be considered annually for patients with IJD and that predicted risk of CVD by established CVD risk algorithms should be adapted with a 1.5 multiplication factor for RA patients fulfilling certain criteria (33). Whether performance of CVD risk evaluation should be the responsibility of the rheumatologist or other health care providers such as general practitioners, specialists in internal medicine, or cardiologists have been discussed (4) and will probably depend on the health care system and economic priorities in each country. However, it may be argued that the responsibility to ensure that a CVD risk evaluation is performed should lie with the rheumatologist, since many patients with IJD are mainly in contact with the health care system in relation to their joint disease (32).

In conclusion, the presence of chest pain was surprisingly infrequently reported in patients with IJD referred for a CVD risk evaluation. However, when chest pain was present, it was weakly associated with CA, in contrast to the predicted CVD risk by several risk calculators, which was highly associated with the presence of CA. These findings suggest that clinicians treating patients with IJD should be alert of coronary atherosclerotic disease also in the absence of chest pain symptoms.

## Author Contributions

All authors have contributed substantially to conception or design of the work; the acquisition, analysis, and interpretation of data; drafting or revising the work critically for important intellectual content; and approval of the final version of the manuscript and agreement to be accountable for all aspects ensuring that questions related accuracy or integrity of any part of the work are appropriately investigated and resolved.

## Conflict of Interest Statement

Silvia Rollefstad has received speaker honoraria from UCB, MSD, and BMS; Eirik Ikdahl has received speaker honoraria from Pfizer; Jonny Hisdahl has no disclosures; and Tore Kristian Kvien has received fees for speaking and/or consulting from AbbVie, BMS, Celgene, Celltrion, Eli Lilly, Hospira, Merck-Serono, MSD, Orion Pharma, Pfizer, Roche, Sandoz, and UCB and received research funding to Diakonhjemmet Hospital from AbbVie, BMS, MSD, Pfizer, Roche, and UCB. Terje Rolf Pedersen has received speaker’s honoraria and consulting fees from Pfizer and Merck/Schering Plough and speaker honoraria from AstraZeneca. Anne Grete Semb has received speaker’s honoraria form Merck/Schering Plough, AbbVie, BMS, and Wyeth and received speaker’s honoraria and consulting fee from AbbVie and Hoffmann LaRoche/Genentech.
